# PARP inhibitor resistance in breast and gynecological cancer: Resistance mechanisms and combination therapy strategies

**DOI:** 10.3389/fphar.2022.967633

**Published:** 2022-08-25

**Authors:** Nannan Wang, Yan Yang, Dongdong Jin, Zhenan Zhang, Ke Shen, Jing Yang, Huanhuan Chen, Xinyue Zhao, Li Yang, Huaiwu Lu

**Affiliations:** ^1^ Department of Obstetrics and Gynecology, The Third Affiliated Hospital of Zhengzhou University, Zhengzhou, China; ^2^ Department of Biochemistry and Molecular Biology, School of Basic Medical Sciences, Xinxiang Medical University, Xinxiang, China; ^3^ Zhengzhou Key Laboratory of Endometrial Disease Prevention and Treatment, Zhengzhou, China; ^4^ Department of Gynaecological Oncology, Sun Yat Sen Memorial Hospital, Guangzhou, China

**Keywords:** PARP inhibitor, PARP inhibitor resistance, breast cancer, gynecological cancer, combination therapy, ATR/CHK1/WEE1 pathway, targeted drugs

## Abstract

Breast cancer and gynecological tumors seriously endanger women’s physical and mental health, fertility, and quality of life. Due to standardized surgical treatment, chemotherapy, and radiotherapy, the prognosis and overall survival of cancer patients have improved compared to earlier, but the management of advanced disease still faces great challenges. Recently, poly (ADP-ribose) polymerase (PARP) inhibitors (PARPis) have been clinically approved for breast and gynecological cancer patients, significantly improving their quality of life, especially of patients with BRCA1/2 mutations. However, drug resistance faced by PARPi therapy has hindered its clinical promotion. Therefore, developing new drug strategies to resensitize cancers affecting women to PARPi therapy is the direction of our future research. Currently, the effects of PARPi in combination with other drugs to overcome drug resistance are being studied. In this article, we review the mechanisms of PARPi resistance and summarize the current combination of clinical trials that can improve its resistance, with a view to identify the best clinical treatment to save the lives of patients.

## 1 Introduction

According to statistics, in 2020, breast cancer had the highest incidence among women in the world, followed by cervical cancer (CC), endometrial cancer (EC), and ovarian cancer (OC) (Sung et al., 2021; Grossman et al., 2018). These cancers seriously endanger women’s physical and mental health. Treatment methods for the cancers mentioned above generally include radical surgical resection, radiotherapy, and chemotherapy (Brown et al., 2020; Butala et al., 2021; Rütten et al., 2021). Although the treatments as mentioned earlier can significantly prolong the survival of patients, the overall prognosis is still unsatisfactory. OC patients have about a 70% probability of recurrence within 3 years after standard treatment (Sung et al., 2021; Ledermann et al., 2018). The 5-years survival rate of early breast cancer patients can reach more than 90%, but the survival rate of advanced cancer patients is only 30%. Compared with the other molecular subtypes of breast cancer, triple-negative breast cancer has the worst prognosis because it does not respond to endocrine or targeted therapy (Sung et al., 2021; Ovcaricek et al., 2011). Advanced EC and CC patients also have a poor prognosis (Cohen et al., 2019; Crosbie et al., 2022). Progression-free survival (PFS) is progressively shorter in patients despite continued treatment at relapse (Poveda et al., 2021).

Recently, the advent of targeted drugs has given new hope to cancer patients, which can prolong the life of these patients, improve their quality of life, and cause less damage to the body. Poly (ADP-ribose) polymerase (PARP) inhibitors (PARPis) are one of these drugs, of which olaparib, lucaparib, niraparib, and talazoparib have been approved for use clinical treatment, including breast, ovarian, lung and prostate cancers (Poveda et al., 2021; Hao et al., 2021; Wang et al., 2020; Mirza et al., 2020; Molinaro et al., 2020). It is the first drug to target and induce the death of BRCA1/BRCA2-deficient cells on a synthetic lethal basis (Bryant et al., 2005). Currently, PARPi has become the first-line regimen for OC treatment (Poveda et al., 2021). A single-center real-world study of patients with platinum-resistant ovarian cancer (PROC) treated with PARPi showed that after the use of PARPi in 17 patients, the objective response rate (ORR) was 47%, and the median progression-free survival (PFS) was 8.2 months (5.3–11.3), overall survival (OS) was 14.9 months (11.2–18.5) (Agarwal et al., 2021). Despite the success of PARPi in targeting BRCA-deficient tumors, the emergence of acquired resistance to PARPi is a hurdle that we need to overcome, with more than two-thirds of patients on long-term PARPi therapy eventually developing acquired resistance (Hodgson et al., 2018; Lin et al., 2019). Therefore, understanding the mechanism of PARPi resistance and then finding alternative treatment strategies to improve the benefits of PARPi treatment is the top priority of our research. This article discusses some of the possible mechanisms of PARPi resistance and combined treatment strategies to improve the sensitivity of PARPi in cancer treatment.

## 2 Mechanisms of PARP inhibitors

There are many mechanisms in cells to recognize and repair DNA damage, so that damaged DNA can be repaired in time to maintain normal physiological functions, mainly including homologous recombination (HR), non-homologous end joining (NHEJ), BER (base excision repair), and mismatch repair (MMR) (Hoeijmakers, 2001). PARP1/2 is crucial in repairing DNA single-strand breaks (SSB) (Langelier et al., 2012). When the DNA single strand breaks, it rapidly recognizes and binds to the DNA damage site, and catalyzes the PARylation of various proteins including itself; i.e., PARP protein and NAD^+^ (Nicotinamide adenine dinucleotide), PAR (poly ADP-ribose) chains bind and recruit other DNA repair-related proteins to initiate the DNA repair process (Fisher et al., 2007; Pascal and Ellenberger, 2015). With the PARylation of PARP1/2 proteins, the chromatin gradually becomes relaxed, and the PARP protein automatically detaches from the DNA damage site. On the one hand, PARPi can block this process, so that the PARP proteins and other DNA repair proteins cannot be detached from the DNA chain (Wang et al., 2019). This is called, “PARP trapping.” This is also the reason why PARPi is more destructive towards cancer cells than knocking out the PARP gene itself (Kim et al., 2020); on the other hand, PARPi can competitively bind to the NAD^+^ site, directly inhibit the activity of PARP1/2, and block the DNA repair process mediated by it, so that the SSB in DNA cannot be repaired and converted into DNA double-strand break (DSB).

**FIGURE 1:**
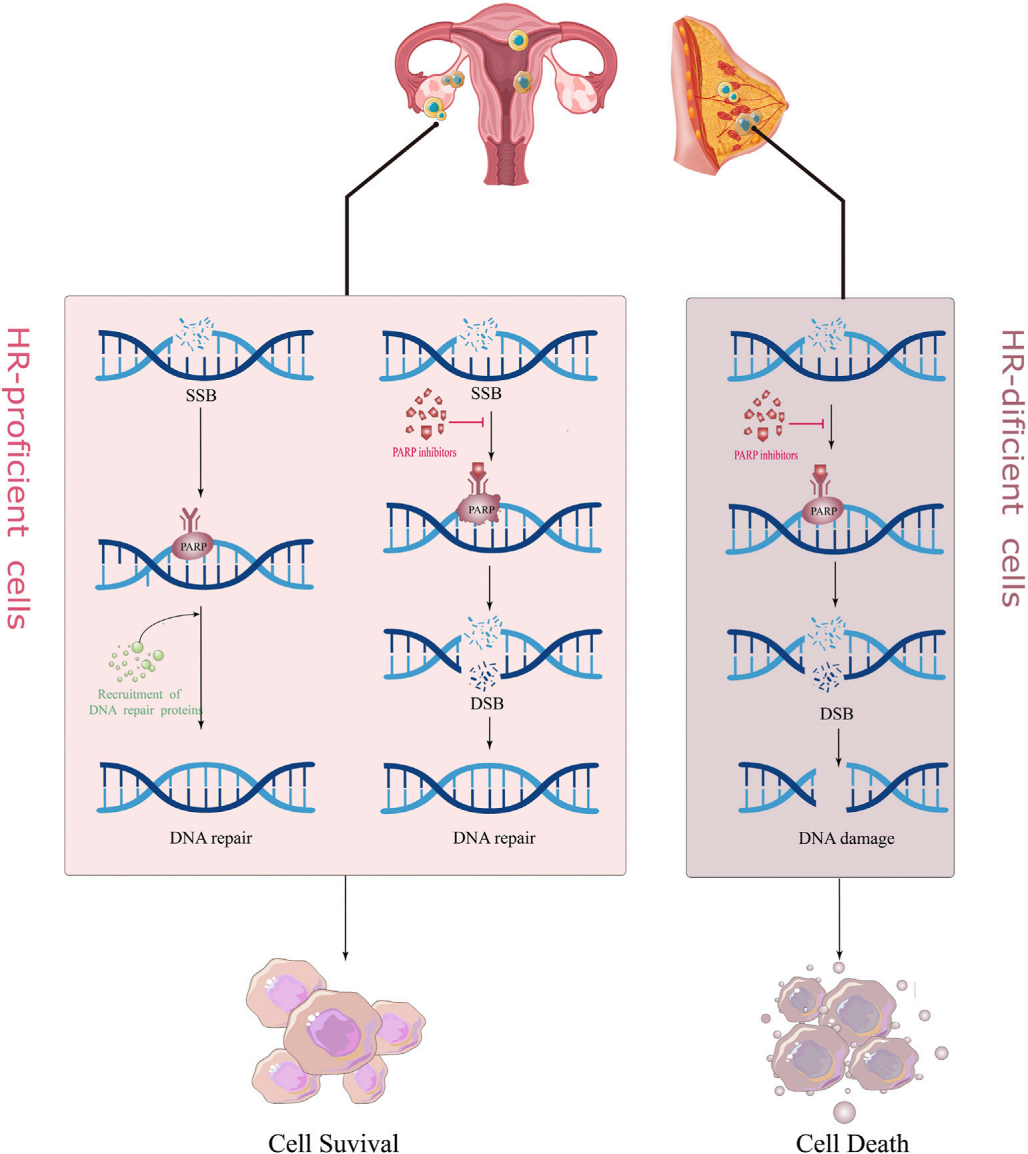
The mechanism of action of PARP inhibitors for “synthetically lethality.”

There are two main repair pathways for DSB, homologous recombination (HR) repair pathway and non-homologous end joining (NHEJ) repair pathway. HR repair (HRR) is a rigorous, high-fidelity repair pathway that contributes to genetic stability. The HRR process involves the participation of various proteins, such as BRCA1, BRCA2, RAD51, and ATM. If the above genes are mutated and lose their original activity, it will lead to homologous recombination deficiency (HRD). At this time, the proportion of NHEJ in the repair process of DSB is increased. Compared with the high fidelity of HRR, the repair of NHEJ does not require the participation of homologous DNA templates; hence, its error rate is extremely high, which easily leads to DNA aberrations and cell death (Betermier et al., 2014; Le Guen et al., 2014). PARPi treatment blocks SSB repair in cancer cells, while cells with BRCA mutations are unable to rely on the HR pathway to repair DSBs and switch to a low-fidelity NHEJ pathway to repair DNA, causing genomic instability and cell death, a so-called “synthesis lethal effect" (Figure 1) (Fishman-Lobell et al., 1992; van Wietmarschen and Nussenzweig, 2018). A recently proposed new theory suggests that in BRCA-deficient cells, PARPi leads to the accumulation of ssDNA gaps through a trans-cellular cycle (Cong et al., 2021). That is, PARPi stimulates the restart of PrimPol to induce the production of ssDNA gaps in S-phase, and the accumulated ssDNA gaps are transformed into DSBs in the next S-phase. The continuous use of PARPi in BRCA-deficient cells leads to the inability of DSBs to be repaired in time and eventually kills tumor cells (Simoneau et al., 2021; Tirman et al., 2021).

## 3 Mechanisms of resistance to PARP inhibitors

With the approval of PARPi as a first-line clinical drug, more and more patients have increased clinical benefits, but with it comes an increase in the incidence of new or acquired resistance to PARPi. An in-depth understanding of the mechanism of PARPi resistance will help us understand and overcome the occurrence of clinical resistance. Numerous preclinical and clinical studies have explored PARPi resistance to identify combination therapy strategies and appropriate populations.

### 3.1 Recovery of replication fork stability

**FIGURE 2:**
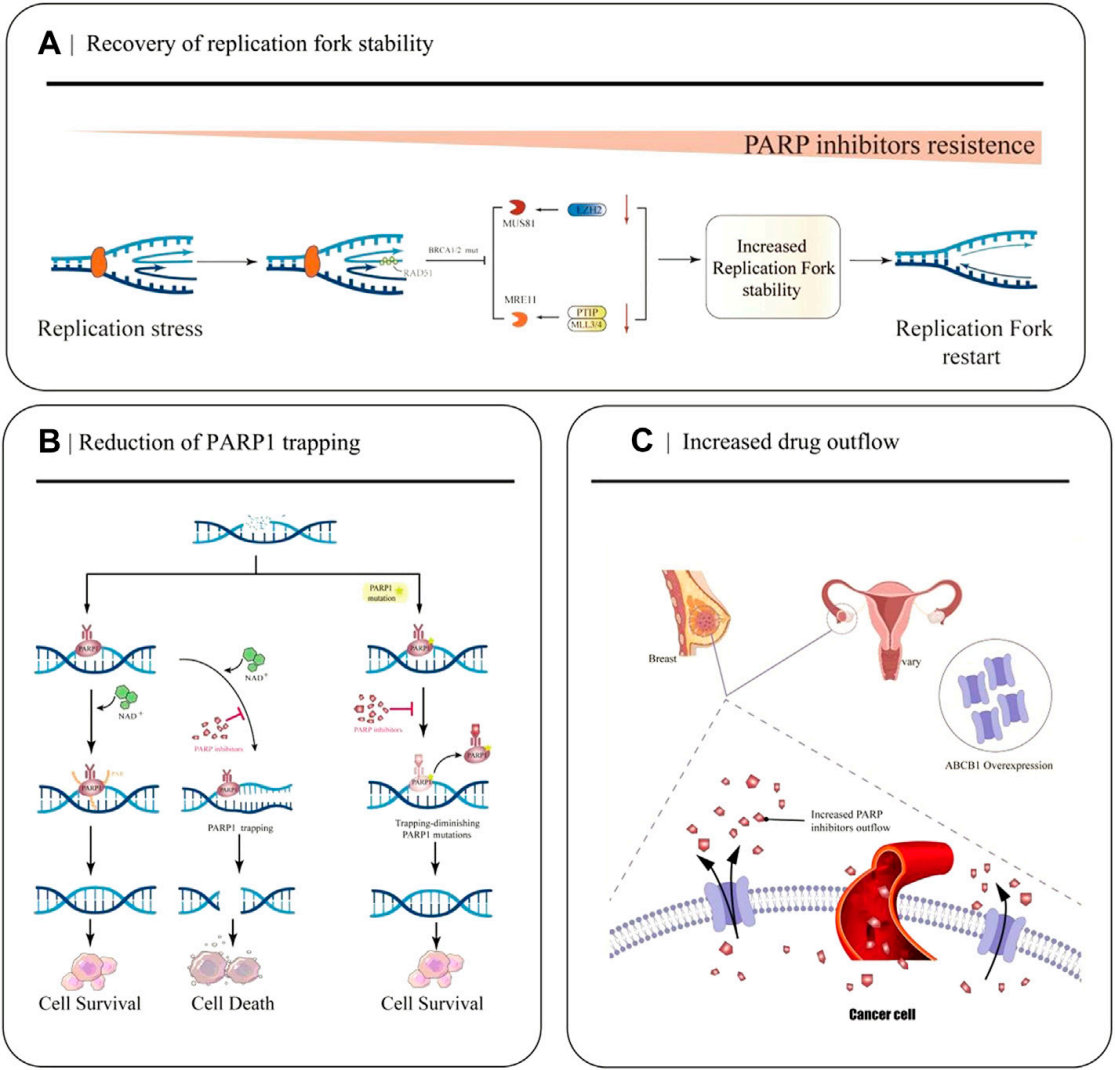
Partial mechanisms of PARP inhibitor resistance in cancer. **(A)** Restoration of replication fork stability leads to PARP inhibitor resistance. When EZH2 or MLL3/4-PTIP is deficient, MUS81 and MRE11 recruitment fails, the replication fork is less attacked, and the replication fork is stable. **(B)** Decreased PARP1 trapping contributes to the development of PARP inhibitor resistance. PARP inhibitors reduce the catalytic activity of PARP1, so that PARP remains bound to DNA and cannot undergo subsequent repair. PARP1 mutations reduce PARP capture. **(C)** Increased drug efflux mediated by ABCB1 overexpression leads to a decrease in effective concentration in cancer cells and increased resistance to PARPi.

The replication fork is a significant step in DNA damage repair and checkpoint activation during replication in the cell cycle. Many abnormalities in DNA replication can lead to stalled replication forks, leading to genomic instability and cell death if stalled replication forks fail to restart (Cox et al., 2000). PARP1, BRCA1/2, and RAD51 exert essential roles in protecting replication forks from nuclease attack and regulating the accumulation of reversed forks (Chaudhuri et al., 2016; Schaaf et al., 2016; Ronson et al., 2018). However, in BRCA-mutated cells, down-regulation of the nuclease MRE11 and cross-linking endonuclease MUS81 due to a series of molecular mechanisms ultimately leads to fork protection and enhanced DNA damage repair (DDR), ultimately leading to PARPi resistance (Figure 2A) (Bryant et al., 2009; Rondinelli et al., 2017).

EZH2, a methyltransferase involved in histone methylation, rapidly locates at the replication fork after replication fork stagnation and promotes MUS81, a crossover junction endonuclease, to attack the replication fork, causing replication fork degradation (Rondinelli et al., 2017). The MLL3/4 complex protein PTIP can recruit the nuclease MRE11 into the stalled replication fork, resulting in degradation of the stalled replication fork (Zhang et al., 2014). In BRCA1 and BRCA2 mutated cells, EZH2 and PTIP activities are downregulated at the fork, and MUS81 and MRE11 recruitment is reduced, ultimately leading to the stabilization of the replication fork and the development of PARPi resistance.

Furthermore, recent studies have found that the lysine acetyltransferase 2B, KAT2B (often called PCAF), is associated with the degradation of stalled replication forks. Kim et al. (2020) showed that in BRCA-deficient cells, PCAF first utilizes its own structure to bind to stalled replication forks, and then acetylates H4K8 to promote the recruitment of MRE11 and EXO1 nucleases to the replication fork, which further promotes fork degradation. Loss of PCAF promotes the resistance of BRCA1/2-deficient cells to PARPi treatment.

### 3.2 Reduction of PARP1 trapping

After identifying DNA damage, PARP1 is rapidly activated and it binds to the site of DNA breakage sites. Then it catalyzes the formation of multiple protein PAR chains, including itself with NAD^+^ as the substrate, thus realizing its function (Wang et al., 2006). PARPi inhibits the catalytic activity of PARP1, which keeps PARP bound to DNA and prevents subsequent repair (Figure 2B). Recently, Stephen et al.used the CRISPR-Cas9 technology to screen PARP1 point mutation fragments that lead to PARPi resistance, and they found PARP1 p. R591C mutation (c.1771C>T) in an olaparib-resistant ovarian cancer patient. Mutations enhance PARP1 dissociation from DNA and reduce PARP trapping, suggesting that PARP1 mutations are associated with the emergence of a drug-resistant phenotype, and experiments have confirmed that point mutations outside the ZnF domain may also lead to PARP inhibition resistance by reducing PARP1 trapping (Pettitt et al., 2018).

### 3.3 Increased drug outflow

Studies have demonstrated that the occurrence of PARPi resistance is associated with increased ABCB1-mediated drug efflux. ABCB1 (P-gp, p-glycoprotein, also known as MDR1), a drug efflux transporter belonging to the ATP-binding cassette (ABC) transporter superfamily, encodes the multidrug resistance protein (Figure 2C) (Li et al., 2015). It has a very wide substrate specificity, and it is highly expressed in many tumors, especially in drug-resistant breast cancer and OC (Christie et al., 2019). With the up-regulation of ABCB 1a/1b genes, the expression of P-gp efflux transporter increases, resulting in a decrease in the effective intracellular drug concentration, thus, leading to PARPi resistance (F. Martins et al., 2021). In 2020, a quantitative mass spectrometry imaging (LC-MS/MS) assay showed that in a P-gp-overexpressing ovarian cancer model, niraparib was unevenly distributed within the tumor, reducing the efficacy of drug treatment; thus, suggesting that it is resistant to PARPi(Morosi et al., 2020).

The relationship between the two was first discovered by Rottenberg et al. The group’s experiments demonstrated that in a breast tumor model, the emergence of PARPi-acquired resistance is related to upregulation of P-gp expression, and that the P-gp inhibitor tariquidar can reverse its overexpression (Rottenberg et al., 2008). Similarly, *in vitro* experiments by Margarida et al. demonstrated that ABCB 1a/1b attenuated brain penetration and promoted systemic elimination of niraparib in mice, with partial reversal of resistance following treatment with the ABCB1 inhibitor Elacridar (F. Martins et al., 2021). A clinical study (NCT02681237) found that ABCB1 was upregulated in 15% of patients in the progression group after using PARPi. Clinically, PARPi resistance is significantly relevant to increased drug efflux and decreased accumulation of PARPis in tumor cells (Lheureux et al., 2020). Therefore, a more in-depth study of the relationship between the expression and role of ABCB1 and PARPi resistance is crucial for clinical medicine.

### 3.4 PARG depletion

**FIGURE 3:**
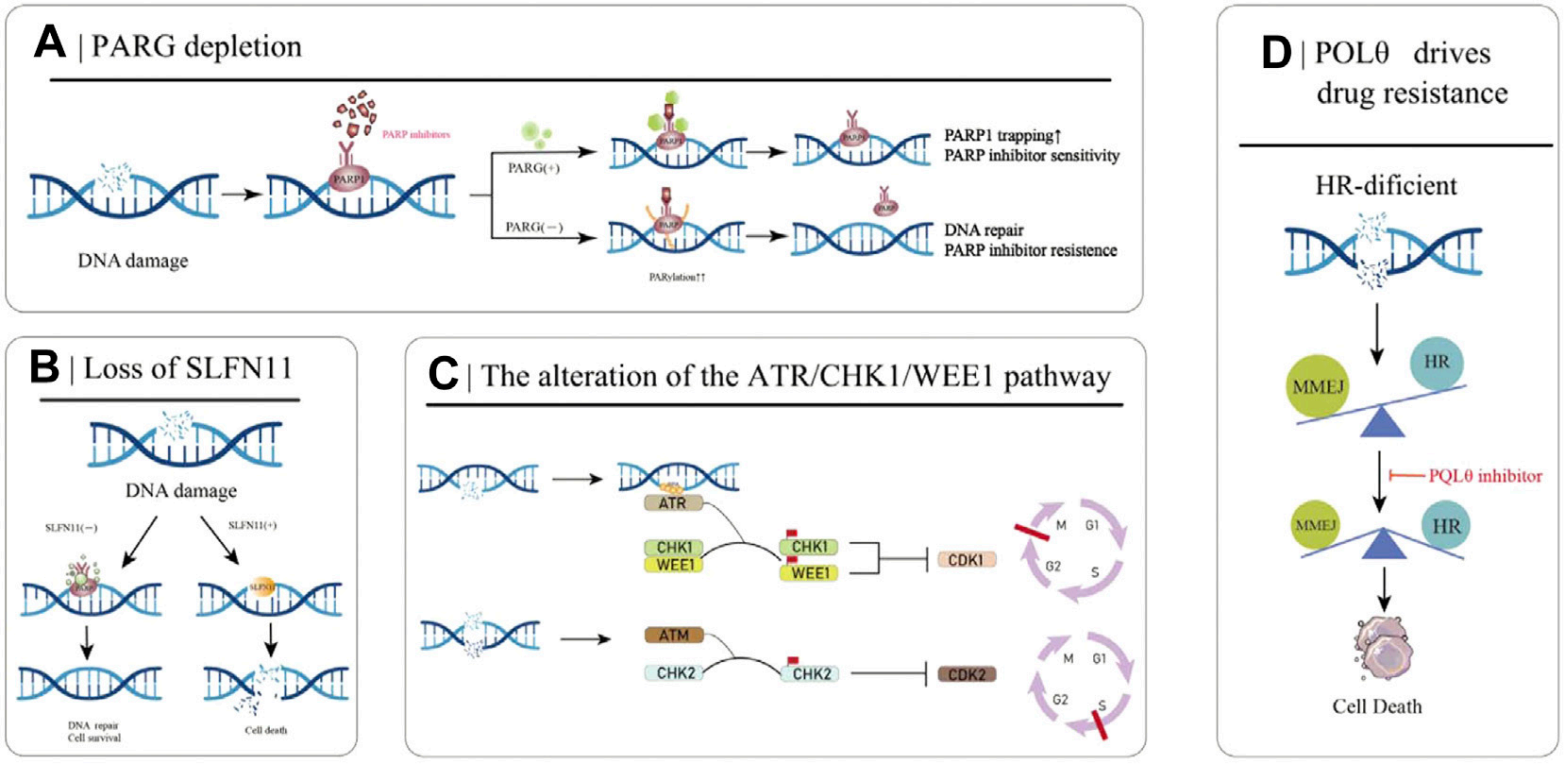
Partial mechanisms of PARP inhibitor resistance in cancer. **(A)** PARG deletion leads to PARPI resistance. **(B)** Loss of SLFN11 enhances DSB repair capacity, ultimately leading to PARPi resistance. **(C)** The ATR/CHK/WEE1 signaling pathway arrests the cell cycle to reduce replication stress and promote DSB repair. **(D)** HR-deficient cells rely on MMEJ for DSB repair, which is mediated by POLθ. Inhibition of POLθ in HR-deficient cells results in cell death.

PARylation is a PARP-mediated post-translational modification of proteins that controls key mechanisms such as the DNA damage response in cells (O’Sullivan et al., 2019). PARP1 is the primary target of PARylation, and the resulting PAR chains recruit downstream protein repair factors. Poly (ADPribose) glycohydrolase (PARG) can reverse PARylation and catalyze PAR strand breaks; thus, its loss reduces PARP1 capture, rescues PARP1-dependent DNA damage signaling, and ultimately leads to PARPi resistance (Figure 3A) (Francica and Rottenberg, 2018; O’Sullivan et al., 2019). A number of studies have demonstrated that the loss of PARG is related to the occurrence of acquired resistance to PARPi. Experiments in breast cancer cell models have demonstrated the correlation between the two (Gogola et al., 2018). Interestingly, many studies of PARPi-resistant cells treated with PARG inhibitors have shown that PARG inhibitors can improve the development of PARPi resistance (Gogola et al., 2018). The PARG protein was highly expressed in 34% of OC tumors, and the use of PARG inhibitors resulted in decreased cell migration compared with the use of PARG inhibitors alone. Inhibition of PARG enhances the therapeutic effect of cisplatin and PARPi on ovarian cancer cells (Chen and Yu, 2019).

### 3.5 Loss of SLFN11

The SLFN11 protein encoded by the Schlafen11 (SLFN11) gene can cause the degradation of specific tRNAs and then inhibit the expression of DNA damage repair-related proteins when activated. Therefore, in normal cells, SLFN11 inhibits DNA repair when DNA is broken. However, the SLFN11 gene is generally silenced in cancer cells, making these cancer cells more capable of DNA repair and conferring resistance to PARPi (Figure 3B) (Li et al., 2012). Loss of SLFN11 conferred resistance to three PARPis (olaparib, rucaparib, and veliparib) in an *in vitro* assay in small cell lung cancer and a dataset analysis in 2017 (Lok et al., 2017). The EVOLVE clinical trial (NCT02681237) exploratory study of anti-angiogenic drugs combined with PARPi in patients with PARPi-resistant ovarian cancer showed the following findings: BRCA1/2 or RAD51B reversion (19%) at the time of PARPi progression; ABCB1 upregulation (15%); and down-regulation of SLFN11 (7%) (Lheureux et al., 2020).

### 3.6 The alteration of the ATR/CHK1/WEE1 pathway

Besides PARPi, the ATR/CHK1/WEE1 pathway also plays a decisive role in DNA damage recognition and repair. When sensing DNA damage and replication fork pressure, replication protein A (RPA) binds to the site of damage and then recruits and activates Ataxia-telangiectasia and RAD3-related protein (ATR) at the site. When ATR is activated by RPA, it starts to activate its effector proteins, CHK1 and WEE1, which eventually leads to cell stagnation in the G2-M and S phases, preventing the cells from entering mitosis to reduce the replication pressure and trigger the appropriate DNA repair pathways (Figure 3C) (Petermann and Caldecott, 2006; Yekezare et al., 2013). PARPi induces strong replication stress after cancer treatment, which activates the ATR/CHK1/WEE1 pathway. Studies have shown that the ATR/CHK1/WEE1 pathway can promote DNA repair, and inhibition of this pathway can resensitize tumor cells to PARPi (Kim et al., 2017)^.^

Cell cycle protein-dependent kinase (CDK) mainly regulates cell cycle and gene transcription process. The expression of DNA repair proteins such as ATR and BRCA1/2 is downregulated by CDK12 knockdown (Bajrami et al., 2014; Naidoo et al., 2018). Inhibition of CDK12 induces the generation of BRCAness phenotype, thus reversing PARPi drug resistance.

### 3.7 POLQ drives drug resistance

Microhomology-mediated end joining (MMEJ) is an uncommonly used DSB repair pathway other than HR and NHEJ, driven by low-fidelity DNA polymerase theta (Polθ, also known as POLQ). It recognizes 5-25bp homologous sequences and directly connects the broken DNA ends. Due to the frequent deletion and dislocation of homologous fragments, MMEJ is a low-fidelity DSB repair pathway compared to HR (Chang et al., 2017). In HR-deficient cancer cells, DSB repair is dependent on MMEJ repair, and inhibition of this pathway results in failure of DNA to repair properly and increases genomic instability (Figure 3D). The expression of Polθ is negatively correlated with HR activity, and Polθ binds to RAD51 and inhibits its mediated DNA recombination. Knockdown of Polθ in HR-proficient cells resulted in increased HR activity and upregulation of RAD51 activity, whereas knockdown of Polθ in HR-deficient cells resulted in cell death (Ceccaldi et al., 2015; Mateos-Gomez et al., 2015). Therefore, on the one hand, inhibiting the Polθ gene in HR-deficient tumors induces tumor cell death through a “synthetic lethal” mechanism, and on the other hand, targeting PARPi-resistant cells caused by 53BP1/Shieldin complex deficiency, is a promising strategy for the treatment of PARPi-resistant patients.

### 3.8 Restoration of homologous recombination repair

**FIGURE 4:**
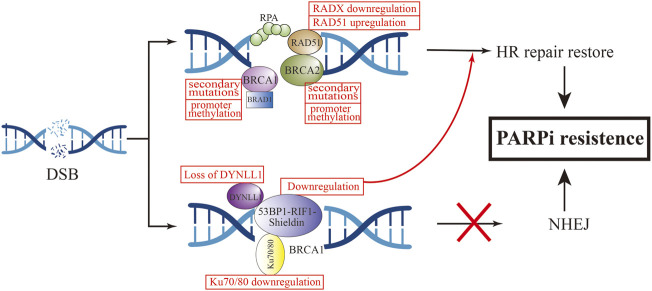
Mechanism of HR-dependent pathway leading to PARPi resistance.

With the approval of PARPi as a first-line clinical drug, more and more patients have obtained increased clinical benefits, but with it comes an increase in the incidence of new or acquired resistance to PARPi. An in-depth understanding of the mechanism of PARPi resistance will help us understand and overcome the occurrence of clinical resistance (Figure 4). Numerous preclinical and clinical studies have explored PARPi resistance to identify combination therapy strategies and appropriate populations.

#### 3.8.1 Restoration of the BRCA1/2 function

To our knowledge, BRCA1 and BRCA2 are critical genes in HR-dependent DSB repair, and the loss of their function can directly lead to defects in the HR-repair pathway (Wang and Figg, 2008). The most common cause of functional restoration of the HR pathway is secondary mutations in BRCA1/2 genes (Barber et al., 2013; Waks et al., 2020). Reverse mutations of BRCA1 and BRCA2 have been demonstrated in high-grade OC cells that are resistant to platinum and PARPi drugs (Shroff et al., 2018). This is due to the reverse mutation correcting the ORF of the primary mutant BRCA1/2. For example, in Ashkenazi Jewish populations, which lack wild-type BRCA2, but carry the c.6174d frameshift mutation of BRCA2, this mutation can result in truncation of BRCA2 protein expression (Edwards et al., 2008). Subsequent further cellular experiments have found that the deletion of the c.6174d mutation and subsequent restoration of the ORF function leads to the expression of new BRCA isoforms, resulting in acquired resistance to PARPi. To investigate the relationship between BRCA reverse mutations and PARPi re sistance, an ARIEL2 trial sequenced the circulating cell-free DNA (cfDNA) of 92 patients with BRCA-mutated OC before and after rucaparib treatment. The PFS of the group with BRCA reverse mutation was 7.2 months less than that of the group without reverse mutation (1.8 vs. 9.0 months, HR, 0.12; 95%CI, 0.05–0.26; *p* < 0.0001) (Swisher et al., 2017).

In addition to reverse mutation of the gene, the reason for restoration of BRCA1/2 function is also related to promoter methylation. Experiments have demonstrated that BRCA1 promoter methylation defects have been detected in some OC ovarian cancer samples. Bianco T et al. observed that unmethylated samples had lower BRCA1 RNA expression levels compared to samples with high BRCA1 methylation. Methylation status of the BRCA1 promoter is associated with BRCA1 silencing, and hypomethylation are correlated with different degrees of BRCA1 repression and different degrees of HR recovery (Bianco et al., 2000). Hence, we can consider that BRCA1 promoter methylation is a useful predictor of the response to PARPi.

#### 3.8.2 Increased expression of RAD51 and secondary mutations in its homologous and paralogous genes

RAD51 is a conserved universal recombinase that forms helical filaments at ssDNA and promotes double-strand repair of broken DNA. On the one hand, it binds to SWI/SNF-related matrix-associated actin-dependent regulator of chromatin subfamily A-like protein 1 (SMARCAL1) at stalled replication forks to repair replication forks and promote fork inversion (Baumann and West, 1998; Arnaudeau, 2001). On the other hand, RAD51 protects newly synthesized DNA from nuclease degradation and pro. motes subsequent DNA synthesis (Hashimoto et al., 2010).

Detecting the expression of RAD51 foci in OC patient-derived xenografts and scoring HR status according to BRCA1/2 status showed that the expression of RAD51 foci was strongly negatively correlated with olaparib responsiveness (Guffanti et al., 2022). In olaparib-treated tumor models, the percentage of RAD51-positive cells was 1.25 ± 0.25% in the four PARPi-sensitive models and 66.54 ± 2.70% in the 14 PARPi-resistant models (*p* < 0.0001). It shows that the high expression of RAD51 is related to the occurrence of PARPi resistance (Castroviejo-Bermejo et al., 2018). Clinical studies have further confirmed the relationship between RAD51 lesions and PARPi. Tumor samples from ovarian patients were stained and scored for RAD51. The results showed that the RAD51 score of PARPi-resistant tumor samples was higher than that of PARPi-sensitive samples, and was negatively correlated with the clinical efficacy of PARPi (Cruz et al., 2018). Similarly, four of eight patients with metastatic BC carrying BRCA1/2 mutations who were treated with PARPi or platinum developed BRCA reversal mutations, and a significant increase in RAD51 expression after drug resistance was found by detecting changes in protein expression in tumor tissues before and after drug resistance (Waks et al., 2020).

It has been confirmed that in addition to BRCA1/2 mutations, RAD51 secondary mutations can also lead to the occurrence of PARPi resistance. Experiments by Olga Kondrashova et al. (2017) showed that mutations in the RAD51C/D genes confer sensitivity to PARPi treatment, but secondary mutations in these genes were detected in ovarian cancer patients who relapsed after rucaparib treatment. It may be that these secondary mutations restore the open reading frame (ORF) of RAD51, thereby restoring HR function and ultimately leading to rucaparib resistance. Further cellular experiments demonstrated that knockdown of RAD51C in the OVCAR8 cell line increased the sensitivity of cisplatin and rucaparib, and reintroduction of wild-type RAD51C cDNA could be observed to restore resistance to PARPi in cells. Conversely, introduction of RAD51C with primary mutation cDNA did not restore resistance. Not only rucaparib, but RAD51C cDNA containing secondary mutations conferred resistance to olaparib, niraparib, tarazopanib, and veliparib. Experiments by Nesic et al. (2021) found that methylation of RAD51 (meRAD51C) sensitizes ovarian cancer models to niraparib and rucaparib treatment, and that if meRAD51C deletion occurs, resistance to PARPi treatment occurs. Recently, a new protein was discovered that can antagonize the activity of RAD51, namely RADX. RADX antagonizes the recruitment of RAD51 at replication forks and inhibits its accumulation at the forks. Conversely, loss of RADX restores replication fork stability, conferring resistance to PARPi in BRCA2-deficient cells. RADX acts as a regulator of RAD51 to regulate stability at replication forks, which can serve as a biomarker for predicting PARPi response (Dungrawala et al., 2017). Additionally, a genome-wide screening found that loss of the ubiquitin ligase HUWE1 induces increased RAD51 expression and promotes partial restoration of HRR in BRCA2-deficient cells, thereby promoting olaparib resistance (Clements et al., 2020).

#### 3.8.3 Defects of non-homologous end joining

In HRD cells, NHEJ plays a major repair role (Betermier et al., 2014; Le Guen et al., 2014). NHEJ does not rely on homologous DNA sequences and directly joins DSB ends by a DNA ligase. But NHEJ is more error-prone than HRR due to deletions or insertions of nucleotide sequences, eventually causing genome instability and ultimately cell death (Grabarz et al., 2012). When the NHEJ regulatory factor is deleted, HR is reactivated, leading to the occurrence of acquired drug resistance.

TP53-binding protein (53BP1) is a protein involved in NHEJ activation, which is normally regulated by BRCA1 to maintain the balance between HR and NHEJ (Bothmer et al., 2010). 53BP1 can protect terminal DNA from excision, thereby inhibiting HR repair (Hong et al., 2016). In BRCA1-mutated cells, loss of 53BP1 restores HR repair by promoting the excision of terminal DNA (Bunting et al., 2010; Chen et al., 2022). Animal experiments demonstrated that knockout of 53BP1 in a mouse breast cancer tumor model with BRCA1 mutations can make PARPi resistant (Jaspers et al., 2013). Decreased expression of 53BP1 was found by whole-exome sequencing and analysis of the HGSOC PDX model with BRCA1 mutations and resistance to olaparib (Parmar et al., 2019). In OC patients with HRD, low expression levels of 53BP1 were associated with suboptimal response to PARPis (Hurley et al., 2019). These *in vitro* and *in vivo* studies have demonstrated that 53BP1 deletion specifically mediates PARPi resistance in BRCA1-mutated tumors. Interestingly, 53BP1 does not appear to mediate PARPi resistance in BRCA2-mutated tumors (Bouwman et al., 2010; Yang et al., 2017).

In the mechanism of mediating HR repair, 53BP1 does not act independently, and the 53BP1-Rap interacting factor (RIF1)-Shieldin axis cooperates with each other in this process (Mirman and de Lange, 2020). 53BP1 controls DNA 5′ excision through RIF1, and REV7 inhibits RIF1 expression (Zimmermann et al., 2013). The Shieldin complex consists of the regulator of viral particle protein expression (REV7), SHLD1, SHLD2, and SHLD3. This complex interacts with 53BP1 to protect DNA ends from excision, promote NHEJ activity, increase the error rate of DNA repair, and promote tumor cell death (Xu et al., 2015; Noordermeer et al., 2018). PARPi resistance also develops when effector expression on this axis is reduced. Dev et al. (2018) found that SHLD1/2 can antagonize the effect of HR, and its reduction is associated with acquired resistance to olaparib.

In addition to the above mechanisms, a novel 53BP1 interactor, DYNLL1, was recently discovered using CRISPR knockout screening technology (He et al., 2018). In BRCA1-deficient tumor cells, DYNLL1 promotes 53BP1 oligomerization to stimulate NHEJ (Becker et al., 2018), and through its interaction with MRE11 Inhibition of DNA end excision (He et al., 2018), the combination of these two effects leads to genomic instability. Low PFS was found to be significantly associated with reduced DYNLL1 expression in patients with BRCA1-mutated OC following chemotherapy. Cellular experiments demonstrated that up-regulation of DYNLL1 expression could reverse the resistance of tumor cells to olaparib. The role of DYNLL1 in DNA repair appears to be effective only in BRCA1-deficient cells, not BRCA2.

Several other recent studies have revealed the role of Ku 70/80 and EZH2 in regulating NHEJ activity in tumor cells. In BRCA-mutated breast and ovarian cancer cell lines, overexpression of miRNA-622 renders them insensitive to olaparib and veliparib treatment. Yang et al. proposed the following mechanism by which miR622 desensitizes PARPi: it downregulats the expression of Ku 70/80, thereby reducing NHEJ activity and restoring HR activity (Choi et al., 2016). EZH2 regulates NHEJ expression in OC cells with high expression of coactivator-associated arginine methyltransferase 1 (CARM1) independent of HR. This study demonstrated that tumor cells with high EZH2 expression were not sensitive to PARPi treatment, and in patient-derived xenografts, EZH2 inhibitors sensitize CARM1-high OCs to PARPi (Karakashev et al., 2020).

The 53BP1-RIF1-Shieldin axis, Ku70/80 and EZH2 can all regulate the expression of NHEJ. Among them, 53BP1 can antagonize the effect of HR through two pathways, namely BRCA1-dependent and BRCA1-independent, which further indicates that PARPi acquired drug resistance. The occurrence is not caused by a single mechanism, and clearer and deeper mechanisms are waiting for us to explore.

## 4 Overcoming resistance to PARPi

**FIGURE 5:**
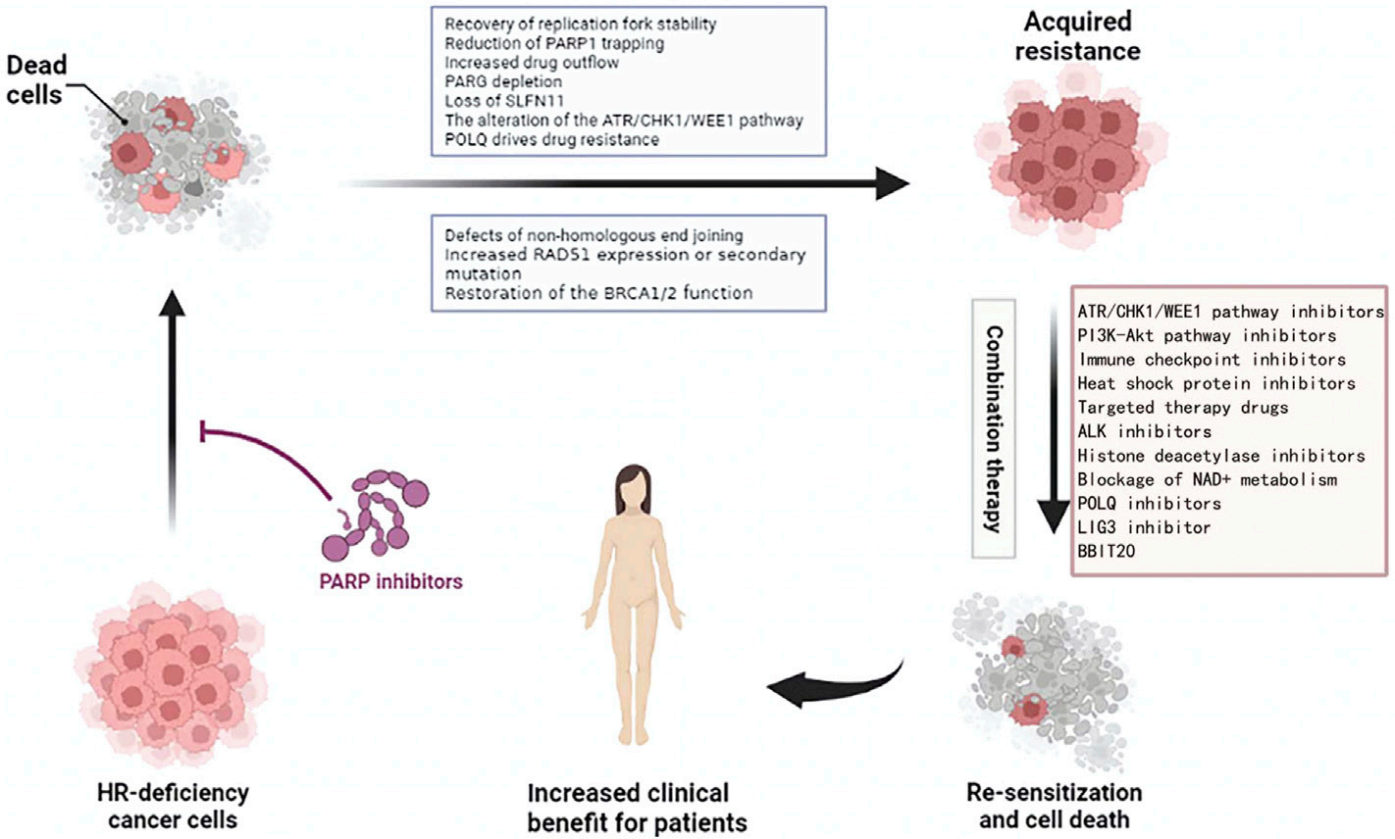
Combination treatment options for ovarian cancer resistant to PARPi. Figures have been created with BioRender.com.

Whether it is congenital or acquired resistance to PARPi, we urgently need new treatments to solve this problem (Figure 5). At present, some clinical trials on the combination of PARPi are under study, and we hope to develop a clinical drug regimen for reversing PARPi resistance.

### 4.1 Combined use of PARPi and the ATR/CHK1/WEE1 pathway inhibitors

Studies have confirmed that drugs, such as ATR inhibitor (AZD6738) and CHK1 inhibitor (MK8776), can reverse the resistance of ovarian cancer cells to olaparib *in vitro*. Interestingly, their combined therapeutic effects with PARPis are independent of HR expression. The effects of ATRi and CHK1i were more pronounced in BRCA-deficient PARPi-resistant cells than in BRCA-proficient cells (Gralewska et al., 2020). This may be because ATR exerts an important role in RAD51 foci formation, and ATRi inhibits the loading of RAD51 onto the stalled forks and causes enhanced fork degradation and eventual cell death. The effect of the combination of these two was also verified in an *in vivo* model. In a mouse ovarian cancer orthotopic model, the use of ATRi resensitized mice to PARPi and increased the sensitivity of PARPi and platinum-based drugs (Kim et al., 2020). A Phase II clinical trial of ceralasertib (ATRi) as monotherapy or in combination with olaparib in ARID1A deletion or non-deletion gynecological cancers (including ovarian, endometrial, cervical, etc.), and treatment outcome will be measured in RECIST v1.1 (Banerjee et al., 2021). In breast cancer, concomitant administration of a CHK1 inhibitor and olaparib restored the sensitivity of BRCA1-deficient resistant triple negative breast cancer (TNBC) cells (Moustafa et al., 2021).

A phase II clinical trial (NCT03579316) compared a WEE1 inhibitor (adavotinib) with or without olaparib in patients with recurrent PARPi-resistant ovarian cancer. The ORR for monotherapy and combination therapy was 23% vs. 29%, and the median PFS was 5.5 vs. 6.8 months. However, grade 3/4 toxicities occurred in both groups, most commonly neutropenia and thrombocytopenia, and these toxicities could be controlled by interruption of treatment or dose reduction (Westin et al., 2021).

Dinaciclib is an inhibitor of CDK1, CDK2, CDK5 and CDK9, and also has some inhibitory activity against CDK12. In BRCA mutant triple negative breast cancer (TNBC) cells, the use of CDK12 inhibitors restored the sensitivity of tumor cells to PARPi, and this effect was also observed in a patient-derived PDX model (Johnson et al., 2016).

The ongoing or completed clinical trials (https://clinicaltrials.gov/) of ATR/CHK1/WEE1 pathway inhibitors combination with PARPi are summarized in Table 1.

**TABLE 1:** Ongoing or completed clinical trials of ATR/CHK1/WEE1 pathway inhibitors combination with PARPi.

	Clinicaltrials. Gov registration/Study name	Phase	Condition or disease	Patients (n)	Combination	Results
Ongoing	NCT04267939	Ib	Advanced solid tumors, including PARP inhibitor resistant OC	56	BAY1895344 (ATRi) + Niraparib	MTD and/or RP2D Incidence of TEAEs Severity of TEAEs-DLT
	CAPRI/NCT03462342	II	ROC (platinum-sensitive or platinum-resistant)	86	AZD6738 (ATRi) + Olaparib	Incidence of TEAEs RRPFS
	NCT04149145	I	PARP inhibitor resistant recurrent OC	40	M4344 (ATRi) + Niraparib	Percentage of patients with TEAEs MTD ORR PFS
	NCT04065269	II	Gynaecological Cancers with ARID1A Loss or no Loss	40	AZD6738 (ATRi) + Olaparib or AZD6738 alone	ORR (complete or partial response)
	NCT03579316	II	PARP inhibitor resistant recurrent OC	104	AZD1775(WEE1i) + Olaparib or AZD1775 alone	ORR DCR
Completed	NCT02723864	I	Refractory solid tumors	53	M6620 (ATRi) + Veliparib + Cisplatin	Incidence of adverse events PR:13.6%
	NCT03057145 Do et al. (2021) PMID: 34131002	I	Advanced solid tumors, including HGSOC with BRCA1/2 mutation	29	Prexasertib (CHKi) +Olaparib	MTD: prexasertib 70 mg/m2 iv and olaparib 100 mg, bid BRCA1mut, PARPi resistant, HGSOC (N = 18): PR 22.22%

DCR-disease control rate; DLT-dose limiting toxicities; MTD-maximum tolerated dose; ORR-overall response rate; OS- overall survival; PFS- progression free survival; RFS- relapse-free survival; RR-response rate; TEAEs- treatment emergent adverse events.

### 4.2 Combined use of PARPi and the PI3K-Akt pathway inhibitors

The phosphoinositide 3-kinase/Akt (PI3K/Akt) signaling pathway regulates many key processes in tumorigenesis and development (Lorusso, 2016). The PI3K/Akt pathway inhibitors have been confirmed to make cancer cells sensitive to PARPi by down-regulating HRR (Mukhopadhyay et al., 2019). Preclinical studies have shown that in TNBC models, the use of PI3K inhibitors down-regulates BRCA1/2, thus, down-regulating HRR and sensitizing cancer cells to PARPi (Park et al., 2013). Combined application of these two is the focus of the current research (Table 2).

**TABLE 2:** Phase II clinical trials of PARPis plus other drugs in OC/TNBC with published results.

Clinicaltrials. Gov identifier/Study name	Condition or disease	Treatment arm	Patients	Key outcome measures
NCT02734004/MEDIOLA	BRCA1/2-mutated metastatic breast cancer	durvalumab + olaparib	30	DCR of 12 weeks: 80% (*N* = 24), 90% CI, 64.3–90.9
NCT03579316	Recurrent PARPi-resistant ovarian cancer	adavosertib	35	ORR: 23% (90% CI)
				CBR: 63% (90% CI)
				PFS: 5.5 months (90%CI)
		Adavosertib + olaparib	35	ORR: 29% (90% CI)
				CBR: 89% (90% CI)
				PFS: 6.8 months (90%CI)
NCT02657889	Triple-negative Breast Cancer	Pembrolizumab + niraparib	55	ORR:21%, (90% CI, 12%–33%)
				DCR:49%, (90% CI, 36%–62%)
				CR:5 patients (9%)
				PR: 5 patients (9%)
				SD: 13 patients (24%)
	Ovarian cancer		60	ORR: 18% (90% CI, 11%–29%)
				DCR: 65% (90% CI, 54%–75%)
				CR:3 patients (5%)
				PR: 8 patients (13%)
				SD: 28 patients (47%)
NCT02354131/AVANOVA2	Platinum-sensitive recurrent ovarian cancer	Niraparib plus bevacizumab	48	PFS:11·9 months (95% CI 8·5–16·7)
		Niraparib	49	PFS: 5.5 months (95% CI 8·5–16·7)

For example, PI3K inhibitors, NVP-BEZ235 and VS-5584, down-regulate HRR and make OC cells with BRCA1 mutations and recombination sensitive to PARPi (olaparib and rucaparib), and studies have shown that NVP-BEZ235 reduces the expression of RAD51. In a preclinical study in TNBC, the PI3K inhibitor BKM120 significantly reduced proliferation of TNBC cell lines by assisting olaparib to block PARP-mediated DNA SSB repair by inhibiting the expression of PARP1 and PARP2 (Li et al., 2021). In 2017, a phase I dose escalation study on the combination of BKM120 and olaparib in the treatment of high-grade serous OC and BC showed that the combination of BKM120 and olaparib was clinically beneficial (the remission rate in patients with advanced OC was 29%, BC patients with a remission rate of 28%) and can be assured of its safety (Matulonis et al., 2017). Phase 1b trial (NCT01623349) of olaparib and another PI3Kα-specific inhibitor (alpelisib) demonstrated partial responses in 36% and stable disease in 50% of 28 patients with BRCA wild-type OC(Konstantinopoulos et al., 2019). Similarly, the combination of these two drugs has also achieved promising results in TNBC. Among the 17 TNBC patients treated, the ORR was 18%, and 59% had control, and the associated adverse events were hyperglycemia (18%) and rash (12%) (Westin et al., 2021).

Currently, NCT01589861 is undergoing Phase II trials of BKM120 and Lapatinib to determine the clinical effect of the combination. Based on a series of preclinical studies, we have reason to believe that this study will achieve satisfactory results. The combination of Akt inhibitors and PARPis can also increase the sensitivity of cancer cells to PARPi. A clinical trial (NCT02208375) investigated the efficacy of olaparib in combination with an Akt inhibitor (capivasertib) in patients with PARP-resistant recurrent EC, TNBC, and OC -- 5 of the 13 treated patients achieved a clinical benefit in the combination study (Yap et al., 2020). The combined application of these two drugs deserves further clinical exploration to overcome the occurrence of PARPi resistance.

### 4.3 Combined use of PARPi and immune checkpoint inhibitors

Immune checkpoints regulate the breadth and strength of immune responses, leading to immune escape and avoiding damage and destruction of normal tissues by the immune system, at present, more researches are on cytotoxic T lymphocyte-associated protein 4 (CTLA-4) and programmed cell death 1 (PD-1) (Pardoll, 2012). CTLA4 and PD-1 compete with CD28 for binding to B7 molecules on antigen presenting cells (APCs), transmits inhibitory signals to T cells, and inhibit their proliferation and activation (Linsley et al., 1994). Immune checkpoint therapy can block inhibitory checkpoints (eg, CTLA-4, PD-1), thereby restoring efficient T cell function and activating the immune system (Zhang et al., 2021). At present, the use of immune checkpoint inhibitors (ICI) has achieved preliminary results in clinical trials, and the most widely used ICI in clinical practice are CTLA-4 inhibitors and PD-1/PD-L1 inhibitors.

The interaction between tumor immune response-related mechanisms and DNA damage is critical in cancer therapy, and one of the most important pathways is cGAS-cGAMP-STING pathway. PARPis release broken double-stranded DNA after treating tumors and dsDNA stimulates STING upregulation, resulting in the production of type I interferon ϒ and pro-inflammatory cytokines (Ding et al., 2018; Li and Chen, 2018; Shen et al., 2019); that is, antitumor immune responses can be mediated after PARPi treatment. Experiments by Meng J et al. (2021) demonstrated that niraparib-induced DNA damage activates the STING pathway *in vitro* and *in vivo*, and increases the expression of membrane PD-L1 and total PD-L1 in OC cells. After combined use of PD-L1 inhibitor, cancer cells are resensitized to T cell killing through mechanisms such as immunomodulation, so as to enhance the antitumor effect. And there was no notable difference in body weight between mice that who were administered the combined drug and the single drug group, indicating that it is a safe option. Therefore, we have reason to believe that the combination of PARPis and immunomodulators may have a stronger effect on inhibiting tumor growth and blocking immune escape.

There are currently more than 20 clinical trials investigating the effectiveness of ICIs in breast cancer and advanced gynecological tumors, but the results are not satisfactory. The ORR of ICIs alone is less than 10% (Maiorano et al., 2021). Niraparib in combination with pembrolizumab (a PD-1 inhibitor) was evaluated in a Phase I/II TOPACIO trial in the treatment of advanced or metastatic TNBC or recurrent OC. In TNBC, the objective response rate (ORR) was 21% and the disease control rate (DCR) was 49% in 47 curative evaluable populations, and the median duration of response (DOR) was not reached at the time of data cut-off. Among 15 patients with BRCA mutations with evaluable efficacy, the ORR and DCR were 47% and 80%, respectively, and the median PFS was 8.3 months. In the 27 cases of BRCA wild-type with evaluable efficacy and in the cohort with BRCA1/2 mutations, ORR and DCR were 45% and 73%, respectively (Vinayak et al., 2019). Among 60 patients with ovarian cancer whose efficacy could be evaluated, the ORR and DCR were 18% and 65%, respectively. ORRs were consistent in each subgroup according to the sensitivity of platinum chemotherapy, whether bevacizumab was used in the past, whether BRCA had mutation or homologous recombination deficiency (HRD). A phase I/II MEDIOLA trial investigating the effects of treatment with olaparib and durvalumab (a PD-L1 inhibitor) in patients with BRCA-mutant metastatic breast cancer, 80% of 30 eligible and treated patients had disease at 12 weeks was controlled, but this trial did not set up an olaparib-alone arm to investigate whether adding durvalumab would increase the clinical benefit of olaparib (Domchek et al., 2020).

A phase II multicohort trial (NCT02912572) investigating the clinical benefit of avelumab (a PD-L1 inhibitor) versus the PARP inhibitor talazoparib in patients with microsatellite stable (MSS) recurrent/persistent endometrial cancer showed that only 3 1 patient had a partial response (ORR = 8.6%) with a PFS of 25.8% at 6 months (Konstantinopoulos et al., 2020). There are several ongoing clinical trials (NCT03016338, NCT03694262) investigating the clinical benefit of combining ICI and PARPi with other drugs in the treatment of endometrial cancer.

Cytotoxic T lymphocyte-associated protein 4 (CTLA-4) acts as an immune checkpoint and downregulates the immune response. Compared with PD-L1 inhibitors, CTLA4 inhibitors have been less studied. A preliminary preclinical study conducted in a BRCA1-deficient mouse model of OC showed that the long-term survival of mice was improved when they were treated with CTLA-4 inhibitors in a synergistic treatment with PARPi (veliparib) (Higuchi et al., 2015). In a clinical trial (NCT02571725) on treatment of patients with recurrent OC with BRCA deficiency, using olaparib and tremelimumab (CTLA4 inhibitor), the Phase 1/2 experiment is underway (Fumet et al., 2020).

However, it seems that the combined use of ICIs and PARPis has not achieved the expected effect, and most experiments only discuss the effect of combined use without setting a PARPis alone group to compare how much additional clinical benefit can be obtained by adding ICIs. Why did the combination of the two not achieve a more significant therapeutic effect? Jennifer et al. suggested that the reason may be macrophage-mediated immunosuppression. Macrophages have an MI-like anti-tumor phenotype and an M2-like tumor-promoting phenotype (Locati et al., 2020). PARPi treatment induces macrophage differentiation to M2 phenotype under the mediation of sterol regulatory element-binding protein 1 (SREBP1) and promotes the expression of colony-stimulating factor receptor (CSF-1R) required for self-survival, while CSF- 1R-positive macrophages inhibit the function of T cells and promote tumor growth and invasion. Previous experiments have demonstrated that inhibition of CSF-1R reduces the production of the M2 phenotype in macrophages, and tumor growth is suppressed in mice lacking CSF-1R (Denardo et al., 2011). In BRCA1-deficient TNBC mice treated with olaparib alone, the PFS was 63 days, the PFS increased to 82.5 days after the combination of a CSF-1R inhibitor and olaparib, and 80% of the tumors in the combination group were completely eliminated on the 34th day. In addition, it was found that the use of olaparib and CSF-1R inhibitors in the BRCA-proficient mouse tumor model had no significant therapeutic effect, indicating that tumor immunotherapy was related to BRCA status.

Further studies found that the use of SREBP1 inhibitor reversed the high expression of CSF-1R induced by PARP inhibitor. In an aggressive TNBC mouse model, treatment with PARPi + SREBP1 inhibitor + CSF-1 inhibitor significantly improved tumor growth inhibition compared to CSF-1 inhibitor + olaparib (Mehta et al., 2021).

### 4.4 Combined use of PARPi and heat shock protein inhibitors

Studies have shown that heat shock protein 90 (HSP90) is highly expressed in lung cancer, breast cancer, and many other cancers (Katoh et al., 2000; Workman and Powers, 2007). HSP90 stabilizes protein conformation through multi-molecular chaperone complexes, promotes the proliferation and growth of cancer cells, and affects many oncogene-related pathways. HSP90 can restore the HR function of cells and make them resistant to PARP by stabilizing the BRCA terminal domain. Studies have shown that the use of the HSP inhibitor 17-allylamino-17-demethoxygeldanamycin (17-AAG) in many cancers degrades BRCA through the ubiquitin-proteasome pathway, making it vulnerable to PARPi. Resensitization demonstrated that dual-target inhibitors of PARP and HSP90 have stronger selective cytotoxicity against tumors (Stecklein et al., 2012). A current preclinical trial on the combination of HSP90 inhibitor onalespib (at13387) and PARPi in the treatment of ovarian cancer shows that in the mouse OC patient-derived xenograft (PDX) model, onalespib can obtain a good therapeutic effect in patients with acquired PARPi resistance without any obvious toxic and side effects. In the subsequent phase I clinical study, a total of 28 patients with advanced solid cancer were enrolled, of which two patients with BRCA-mutated HGSOC who had previously received Olaparib and onalespib therapy had stable disease after 24 weeks of treatment. Overall, 68% of patients had stable disease, 32% had progressive disease, and 32% experienced clinical benefit from the regimen (Konstantinopoulos et al., 2022). At present, the HSP90 inhibitor TAS-116 (pimitespib) has achieved great results in the treatment of gastric stromal tumors, significantly prolonged PFS compared to the original treatment (Doi et al., 2019; Saito et al., 2020). It is expected that other HSP90 inhibitors will achieve good therapeutic effects in gynecological tumors.

### 4.5 PARPi combined with targeted therapy drugs

The targeted drugs currently studied in combination with PARP for the treatment of cancer mainly include anti-angiogenic drugs (such as bevacizumab) and EGFR-targeted drugs (such as cetuximab).

Anti-angiogenic drugs make tumor cells achieve a hypoxic state by inhibiting tumor angiogenesis, causing DNA damage in tumor cells, and the most characteristic feature is DNA DSB; at this time, DNA replication pressure increases, and then the expression of HRR pathway-related proteins, such as RAD51 and BRCA1/2, is down-regulated through various ways, and the final result is HRR defects. Therefore, in combination with antiangiogenic drugs, it may increase tumor sensitivity to PARPi. Bevacizumab inhibits the vascular endothelial growth factor (VEGF) to inhibit tumor growth and invasion. In the phase II trial of AVANOVA2, one group of patients with recurrent ovarian cancer received niraparib alone and the other group received niraparib and bevacizumab in combination. PFS was significantly improved (11.9 vs 5.5 months, *p* < 0.001). In another clinical study of olaparib combined with cediranib in the treatment of patients with OC, the effect of the combination therapy was stronger than that of monotherapy, especially in the non-BRCA mutation group. The PAOLA phase III trial enrolled 806 patients with advanced high-grade ovarian cancer to receive olaparib or olaparib in combination with bevacizumab. Compared with bevacizumab alone, treatment with PARPi significantly improved the patients’ survival rate. Progression-free survival (22.1 vs. 16.6 months; HR 0.59; 95% CI, 0.49–0.72; *p* < 0.001). Among patients with HRD-positive ovarian cancer, the combination group had the most significant benefit, with a median PFS of 37.3 months in the combination group and 17.7 months in the bevacizumab-alone group (HR, 0.33; 95% CI, 0.25–0.45), among HRD-positive patients without BRCA mutations, median PFS was 28.1 months in combination therapy versus 16.6 months in monotherapy (HR, 0.43; 95%) CI, 0.28–0.66). Adverse events occurred in 31% of patients in both groups, with a higher rate in the bevacizumab plus olaparib group than in the bevacizumab alone group. Olaparib plus bevacizumab as first-line maintenance therapy provided significant antitumor activity and safety in HRD-positive patients regardless of BRCA status (Ray-Coquard et al., 2019).

Cetuximab acts on the epidermal growth factor receptor (EGFR) and blocks the intracellular signal transduction pathway by inhibiting tyrosine kinase (TK) bound to EGFR, thereby inhibiting cancer cell proliferation and inducing apoptosis. *In vitro* experiments on head and neck tumors demonstrate that inhibition of EGFR with cetuximab (C225) sensitizes cells to PARPi (ABT-888) (Nowsheen et al., 2011). This is because C225 reduces NHEJ and HR-mediated DSB repair, resulting in DNA damage that persists after PARPi use. Through this mechanism, C225 can make the head and neck tumor cells susceptible to PARP inhibition. Therefore, the combination of C225 and ABT-888 may be an innovative therapeutic strategy, but this combination method has not been currently used in gynecological tumors, and further research is needed.

### 4.6 PARPi combined with ALK inhibitors

Ceritinib is an ALK kinase inhibitor for first-line ALK-positive treatment-naïve patients. It can inhibit the complex I of the mitochondrial electron transport chain, which cooperates with PARPis to generate reactive oxygen species, and then reactive oxygen species can induce DNA damage (Friboulet et al., 2014). Repair of this damage requires the participation of PARP. These mechanisms suggest that ceritinib combined with PARPis may increase its therapeutic efficacy in cancer. The combination of ceritinib and olaparib showed superior cancer inhibition in OC cell lines, and also in the HGSOC PDX model; the results showed that ceritinib monotherapy had no significant effect, but it could increase the therapeutic effect of olaparib in the PDX model (Kanakkanthara et al., 2022), and this result provides the basis for the clinical study on the combination of these two. There is currently no clinical trial to further explore the effect of the combined use of these two drugs on the clinical benefit of patients, which is also the direction of future research.

### 4.7 PARPi combined with histone deacetylase inhibitors

Histone deacetylase inhibitors (HDACi) can reduce the expression of HRR-related proteins (Roos and Krumm, 2016; Wilson et al., 2022). Vorinostat is the first HDACi to be developed. *In vitro*, transcriptome sequencing results showed that the expression of HR repair-related molecules was decreased in OC cell lines treated with the HDACi panobinostat. Likewise, the combined use of PARPi and HDACi enhanced DNA damage and decreased HR repair capacity (Wilson et al., 2022). *In vivo* experiments showed that HDACi combined with PARPi significantly inhibited the growth of xenograft tumors compared with the two drugs alone--OC and TNBC increased the antitumor effect after treatment with the two drugs (Yalon et al., 2016). Olaparib in combination with Entestat, a novel HDAC inhibitor, is being studied in a phase I/II clinical trial (NCT03924245) in the treatment of relapsed, platinum-refractory or resistant HGSOC and fallopian tube cancer. Key outcome measures are MTD and ORR.

### 4.8 PARPi combined with drugs targeting NAD^+^ metabolism

PARP1 uses oxidized NAD^+^ as a substrate for PARylation, which plays a key role in DNA repair and cell signal transduction. Inhibition of NAD^+^ production modulates PARPi responsiveness. Over-activation of PARP1 caused by oxidative stress and DNA damage can cause a rapid decrease in intracellular NAD^+^ concentration, resulting in a rapid decline in the level of intracellular energy metabolism (Houtkooper et al., 2010). Cells maintain the level of NAD^+^ through the salvage pathway, and nicotinamide phosphoribosyltransferase (NAMPT) exerts its biological function as the rate-limiting enzyme of the NAD^+^ salvage synthesis pathway (Tateishi et al., 2015). Bajrami et al. observed that knockdown of NAMPT in TNBC cell lines increases olaparib sensitivity. Olaparib alone caused a 36% inhibition of cell survival in BRCA-deficient TNBC cell lines, which was increased to 72% when the NAMPT inhibitor (FK866) was added. *In vivo* experiments were carried out in the TNBC model of nude mice, and olaparib, FK866, and olaparib plus FK866 were used to treat xenograft model mice. The results showed that compared with any single drug treatment, the drugs in the combination group significantly inhibited the tumors growth, and two nude mice in the combined group (*n* = 10) had complete tumor regression at 39 days. FK866 can significantly inhibit the growth and proliferation of OC cells both *in vitro* and *in vivo*, but there is no study to explore the effect of combination therapy (Bajrami et al., 2012). In addition, NADP^+^,a derivative of NAD^+^ competes with PARP to bind to the NAD^+^ site and inhibits PARylation. It can be regarded as an endogenous PARP inhibitor. When the NADP^+^ content in cancer cells is high, it will confer PARPis sensitivity (Bian et al., 2019). In conclusion, the relationship between the regulation of NAD^+^ metabolism and PARPi sensitivity is still in its infancy, and more preclinical and clinical studies are needed to explore the link between the two, in order to utilize the metabolic fragility of cancer cells to improve PARPi sensitivity.

### 4.9 PARPi combined with POLQ inhibitor

In addition to being an antibiotic, novobiocin (NVB) can also inhibit the activity of POLQ ATPase. Both *in vivo* and *in vitro* experiments have demonstrated that NVB can induce HR-deficient tumor cell death (Zhou et al., 2021). ART558 is a small-molecule Polθ inhibitor that has a synergistic effect with PARPi, and it has a stronger inhibitory effect on cells (including ovarian cancer and breast cancer cells) when used at the same time in BRCA1/2 mutant cells. Interestingly, tumor cells were more sensitive to POLQ inhibitors when the expression of 53BP1 in BRCA-deficient cells was reduced, suggesting that ART558 may resensitize patients with acquired resistance to PARPi by inhibiting DNA end excision protection (Zatreanu et al., 2021). ART4215 is the first Polθ inhibitor to enter clinical development and is currently undergoing clinical trials of ART4215 as monotherapy or in combination with PARPi inhibitors in advanced solid tumors, and treatment is still ongoing. POLQ inhibitors are promising drugs either as an alternative to or in combination with PARP inhibitors.

### 4.10 PARPi combined with LIG3 inhibitor

Single-stranded DNA gaps are created after PARPi treatment, and DNA ligase III (LIG3) is a known DNA repair factor that catalyzes DNA end joining (Taylor et al., 2000). Inhibition of LIG3 in BRCA1-deficient cells and in BRCA1/53BP1 double-deficient cells promotes PARPi-induced accumulation of single-stranded DNA gaps, ultimately leading to accumulation of chromosomal aberrations and cell death. Experiments showed that compared with parental cells, the sensitivity of tumor cells to PARPi was significantly increased after LIG3 knockout; PARPi-resistant cells regained sensitivity to PARPi after LIG3 depletion. Reversal of PARPi resistance by LIG3 is due to increased single-stranded DNA gap independent of HR (Paes Dias et al., 2021). The combined effect of the two needs further animal experiments and clinical trials to verify.

### 4.11 PARPi combined with BBIT20

In addition to the above chemical drugs, a natural monoterpene indole alkaloid derivative, namely dregamine 5-bromo-pyridin-2-ylhydrazone (BBIT20), has also made new progress in reversing PARPi resistance. BRCA1-associated ring domain protein (BARD1) binds to BRCA1 to form BRCA1-BARD1, which can self-localize to DSB and promote HR-mediated DSB repair. BBIT20 can inhibit the interaction between BRCA1 and BARD1, resulting in decreased BRCA1 expression, especially in nuclear BRCA1, thereby inhibiting HRR. BBIT20 significantly enhanced olaparib in patient-derived OC cells and TNBC cells without inducing drug resistance. BBIT20 and olaparib were then used to treat OC xenograft mouse models, respectively, and the results showed that BBIT20 inhibited tumor growth more strongly than olaparib (Raimundo et al., 2021). These preclinical studies show that BBIT20 has a very promising application in the treatment of cancer, whether as a single drug or a combination drug.

## 5 Conclusion and future outlook

An increasing number of preclinical and clinical studies help us understand the mechanism of PARP inhibitor resistance. At present, the use of PARPis as first-line maintenance therapy for breast and ovarian cancer is satisfactory, and its clinical application range is gradually expanding (such as pancreatic cancer and prostate cancer). However, there are many troublesome problems behind the surprise of PARPis, and the occurrence of acquired drug resistance is one of the problems that cannot be ignored. The development of PARPi resistance, as described in this paper, is caused by a variety of mechanisms, most of which have been studied in cell lines and mouse models, but the actual clinical treatment is different from pure preclinical research. Many patients have developed resistance to drugs, such as platinum or paclitaxel, before using PARPi treatment, which will lead to the generation of PARPi cross-resistance. Therefore, the resistance of PARPi in clinical treatment may be different from preclinical studies. More clinical samples and studies are needed to further determine a more comprehensive mechanism of PARPi resistance.

In order to solve the problem of PARPi resistance, we studied the therapeutic effect of PARPis combined with other drugs in order to improve its therapeutic effect, PARPis plus ATR/CHK1/WEE1 pathway inhibitors, PARPis plus PI3K/Akt pathway inhibitors, PARPis plus ICIs, PARPis plus HSP90 inhibitors, PARPis plus targeted therapy drugs, PARPis plus ALK inhibitors, PARPis plus HDACis, PARPis plus drugs targeting NAD^+^ metabolism, and PARPis plus POLQ inhibitor have shown certain effects in preclinical and clinical studies. But with so many types of drugs, which one will bring the greatest benefit to the patient? Will the combination drug produce more toxic and side effects while improving the efficacy and prolong the survival of patients, and will the quality of life of patients decrease? Can patients afford the financial stress of multiple medications? These are hot issues of clinical concern.

According to the existing clinical studies, PARPis combined with the ATR/CHK1/WEE1 pathway inhibitors is the current choice that takes into account the clinical benefits and safety. The patients’ PFS and OS have been significantly improved, and the adverse reactions are also within the range of patients’ tolerance. In recent years, ICIs, as revolutionary drugs for tumor treatment, have made great progress in clinical treatment as a single treatment plan, but unfortunately they have not achieved 1 + 1>2 effect in combination with PARPis; the therapeutic effect of combined targeted drugs is acceptable, but the incidence of adverse reactions in patients is greatly increased. Research on other drugs is currently mostly concentrated in preclinical studies, with no clinical trials or experiments in progress. According to the mechanism analysis, POLQ inhibitor induces tumor cell death from two aspects and seems to be a more promising drug. Finally, further exploration of biomarkers associated with PARPi response and clinical classification based on them to determine which combination therapy is most suitable for patients will allow for more individualized clinical treatment.
